# DeepBehavior: A Deep Learning Toolbox for Automated Analysis of Animal and Human Behavior Imaging Data

**DOI:** 10.3389/fnsys.2019.00020

**Published:** 2019-05-07

**Authors:** Ahmet Arac, Pingping Zhao, Bruce H. Dobkin, S. Thomas Carmichael, Peyman Golshani

**Affiliations:** ^1^Department of Neurology and University of California, Los Angeles, Los Angeles, CA, United States; ^2^Semel Institute for Neuroscience and Human Behavior, University of California, Los Angeles, Los Angeles, CA, United States; ^3^West Los Angeles Veterans Affairs Medical Center, Los Angeles, Los Angeles, CA, United States

**Keywords:** behavior analysis, deep learning, motor behavior, social behavior, human kinematics

## Abstract

Detailed behavioral analysis is key to understanding the brain-behavior relationship. Here, we present deep learning-based methods for analysis of behavior imaging data in mice and humans. Specifically, we use three different convolutional neural network architectures and five different behavior tasks in mice and humans and provide detailed instructions for rapid implementation of these methods for the neuroscience community. We provide examples of three dimensional (3D) kinematic analysis in the food pellet reaching task in mice, three-chamber test in mice, social interaction test in freely moving mice with simultaneous miniscope calcium imaging, and 3D kinematic analysis of two upper extremity movements in humans (reaching and alternating pronation/supination). We demonstrate that the transfer learning approach accelerates the training of the network when using images from these types of behavior video recordings. We also provide code for post-processing of the data after initial analysis with deep learning. Our methods expand the repertoire of available tools using deep learning for behavior analysis by providing detailed instructions on implementation, applications in several behavior tests, and post-processing methods and annotated code for detailed behavior analysis. Moreover, our methods in human motor behavior can be used in the clinic to assess motor function during recovery after an injury such as stroke.

## Introduction

A major goal in neuroscience research is to understand the relationship between neural function and behavior (National Institute of Health BRAIN 2025: A Scientific Vision, 2014). In order to understand this relationship, a vast array of exciting technologies have been developed over the years to characterize the structure and record the activity of neuronal populations (Real et al., 2017), as well as to modulate neuronal activity at cellular resolution and millisecond timescale (Deisseroth, 2015). In contrast, the development of behavioral analysis has lagged, with indirect measurements and a reductionist approach (Krakauer et al., 2017). This is, in part, due to a lack of tools to do automated and detailed analysis of behavior.

Observation and description of natural animal behavior has been fundamental to ethology (Tinbergen, 1963). Although modern high-speed video can record the natural behavior of animals in exquisite detail, analysis of these recordings can be extremely difficult. The blinded observation and description of the video-recordings can take much longer than the time needed to record them, and these observations are highly subjective. Thus, tools that automate the analysis of these videos are needed for faster and more objective description of the video recordings. Several methods have been developed for this purpose: For example, classical machine vision techniques combined with depth imaging can identify patterns of behavior (Wiltschko et al., 2015). However, this requires special depth cameras and is not generalizable to all types of images. Other studies have used machine vision techniques with unsupervised data analysis (Vogelstein et al., 2014; Robie et al., 2017). While unsupervised analysis is very promising to identify patterns inherent to the data, it is not easy to apply the classical machine vision techniques to different behavior/experimental settings. While the commercial systems can provide off-the-shelf solutions for some behavioral tests, they are not open-source, thus limiting any type of modification, and their application to other behavioral tests. Automated tools that can easily be implemented and generalized to many different behavior tests are needed.

Advances in the deep learning field present opportunities for the automated analysis of images (LeCun et al., 2015). More specifically, convolutional neural networks (CNN), a class of deep neural networks, are most commonly used for image analysis. They are made up of nodes (“neurons”) with learnable/trainable weights and biases, and the architecture is comprised of width, height (similar to images) and depth (a third dimension of activation volume) (Lecun et al., 1998). There have been recent advances in the field with several different CNN architectures (Krizhevsky et al., 2012; He et al., 2015; Szegedy et al., 2015) resulting in faster and more accurate outcomes.

Recently, deep learning applications have been used in behavior imaging data analysis (Stern et al., 2015; Mathis et al., 2018; Pereira et al., 2018). The first one of these studies created their own network architecture (Stern et al., 2015) which can limit the implementation of the technique and its broad use. The other two approaches (Mathis et al., 2018; Pereira et al., 2018) showed successful implementation of the CNNs to behavior imaging data analysis, both of them focusing on body pose estimation in animals. One of these used transfer learning approach on only one network architecture (Mathis et al., 2018), whereas the other one trained the network from scratch and achieved similarly good results (Pereira et al., 2018). These two approaches focused on animal pose estimation. While this provides useful information for behaviors where the pose detection of individual body parts is important, it cannot perform direct object recognition (for example distinguishing an apple vs. an orange). Specifically, it cannot distinguish two mice in different positions (vertical vs. horizontal) or identify a mouse performing a specific behavior (such as grooming). Therefore, these networks would detect body positions but not recognize that position/behavior directly. In order to identify these specific behaviors or body positions, these algorithms would require inferences based on the pose coordinates of body parts. Moreover, both studies used only one neural network architecture, thus limiting the user from trying and comparing different network architectures. Additionally, they did not provide post-processing methods for 3D kinematic analysis.

Similarly, motor behavior analysis in humans has also lagged. Currently, the most commonly used clinical motor function assessment tests are based on subjective scoring of the outcome (whether a task is completed fully, partially or not at all). These types of clinical motor impairment scores (i.e., Fugl-Meyer, Action Research Arm Test) are based on ordinal scales, and are insensitive to detect the meaningful changes in the motor function. Moreover, this is important because this type of simple and inexact motor impairment scores or, even worse, disability scores (modified Rankin Score) are not adequate (Bernhardt et al., 2017), and may not accurately reflect true recovery (Kitago et al., 2013). It is important to distinguish between the compensatory movements and true recovery, which can best be done via kinematic analysis (Cirstea and Levin, 2000; Kitago et al., 2013; Krakauer and Carmichael, 2017). Kinematic analysis reveals the timing and typicality of movements, and allows compensatory actions to be distinguished from true recovery of function (Krakauer and Carmichael, 2017). Moreover, it also provides objective metrics that have the potential to capture the movement quality. However, performing kinematic analysis on human motor behaviors can be challenging. Various sensors, reflective markers, external devices, or robotics have been used to perform kinematic analyses (Krebs et al., 2014). The complexity and cost of these devices greatly limits their generalized use. Moreover, using external devices may also alter the natural behavior itself. Thus, marker-less, automated analysis methods are needed for clinical assessment of motor function.

Here, we present a deep learning toolbox and post-processing methods. We name this toolbox DeepBehavior. We expand the deep learning applications for animal behavior imaging analysis by using two different CNN architectures in three different rodent behaviors (food pellet reaching task and two social behaviors). We demonstrate three dimensional (3D), marker-less kinematic analysis of reaching movement in mice. We provide detailed analysis of social behavior when two mice are interacting with post-processing methods. We show evidence that transfer learning approach accelerates training of the network with these types of images. Furthermore, we also demonstrate how CNNs can be used in clinical settings to assess motor function to perform 3D kinematic analysis of motor function in humans.

## Materials and Methods

### Animals

All animal procedures were approved by the University of California, Los Angeles, Department of Laboratory Animal Medicine Institutional Animal Care and Use Committee, and were in accordance with the AAALAC and NIH guidelines. The animals used in this study were either GAD2CrexAi9 or C57Bl6/J mice, and both male and female mice were included. The age range of mice was 10–16 weeks-old.

### Human Subjects

A 35-year-old, healthy adult was recorded. A written informed consent was obtained prior to the recording in accordance with the Declaration of Helsinki. The consent included the use of video recordings for research, education, publication and public presentation.

### Skilled Food Pellet Reaching Test

We have developed an apparatus for head-fixed mice to perform a reaching task for a food pellet. This apparatus is 3D printed (Shapeways, New York, NY) and has an arm that controls a platform with scotch and yoke mechanism. The arm is controlled by a small servo motor (Sparkfun, Niwok, CO). This is connected to a plexiglass cylindrical food pellet dispenser that is controlled by a stepper motor (Sparkfun, Niwok, CO). This releases one food pellet at a time. The apparatus automatically detects the pellet removal with an infrared light sensor, and provides a new pellet in the same exact position. During this time, the animal's paw is video recorded at 124 frames per second by two, monochrome, USB3.0, CMOS cameras (ThorLabs, Newton, NJ) at 448x460 pixel image size. The videos were recorded by using StreamPix software (Norpix, Montreal, QC, Canada) and were saved as “.seq” files. Then, a custom-written Python script was used to generate “.png” images and “.avi” video files from the “.seq” files. The servo and stepper motors and the infrared sensors are all controlled by an Arduino circuit board with a custom designed PCB shield. The cameras were triggered by a function generator (Siglent Technologies, Solon, OH). The animals were trained in this setup for 2 weeks until they were reaching for the food pellets on their own repetitively.

### Three-Chamber Test for Sociability

We have custom built a plexiglass box (60 × 45 × 45 cm) with three chambers divided by plexiglass walls with spaces (45 × 19 × 45 cm) on them to allow exploration. Each side chamber has an upside down wired cup with one of them empty and the other one with a stranger mouse inside. The experimental animal is gently placed in the middle chamber, and is allowed to explore for 10 min. During this time, the whole apparatus is recorded from the top by using a Logitech web camera at 30 frames per second. In some recordings, the mouse wears a miniaturized fluorescence microscope on the head for simultaneous calcium imaging recordings. We calculate the time exploring each cup and their percentages of total time.

### Social Interaction in Home Cage Test

For this test, two mice (one with a miniaturized microscope) were placed in a custom made, 45 × 45 cm plexiglass chamber, and their interaction was recorded from top view by using a monochrome, USB3, BlackflyS camera (Flir, Richmond, BC, Canada) at 30 frames per second.

### Human Motor Behavior Recording System

We have built a stereo camera system with two high speed (170 Hz) color CMOS cameras (Flir, Richmond, BC, Canada). The cameras were fixed (62 inches apart from each other) on a foldable optical aluminum rail (McMaster-Carr) so that their positions and angles were fixed relative to each other. The orientation of cameras was almost orthogonal to each other. The cameras were connected to each other with a general I/O cable to provide synchronization between the cameras, and to a laptop computer with 32GB RAM for data acquisition. SpinView software (Flir, Richmond, BC, Canada) was used to acquire the videos. The aluminum rail that the cameras were fixed on was then placed on a tripod. The videos were recorded at 1,280 × 1,024 pixels resolution and at 170 frames per second. For reaching test, the subject sat on a chair and while sitting straight up reached for a ball hanging from the ceiling. For supination/pronation task, the subject sat on a chair and alternately rotated both hands.

### Converting Videos to Single Frame Images

The Streampix software saves the images in “.seq” format (reaching task). Using Python PIMS (Phyton image sequence) package, and a custom Python script, we convert these video files to folders of images in “.png” format. We then make “.avi” format video by using ffmpeg. To process the “.avi” videos (social behavior), we use ffmpeg.

### Creating Training and Test Datasets

In order to train the neural networks, we used custom written Python scripts to obtain bounding box coordinates for the paw positions. This script creates “.json” files that include x1, x2, y1, and y2 coordinates of the bounding boxes for each image in a folder. A different set of images were also labeled using the same script but then used as a test dataset. These “.json” files and the folders of corresponding raw images are then used as the training and test datasets for the GoogLeNet network (Stewart et al., 2016). This network model was written in Python and Tensorflow (Google) framework. We determined the size of the training dataset as described in the Results section. Because there is only one bounding box to be labeled, the labeling process is rather fast (we were able to label 100 images in ~20 min). For the two-mouse interaction assay, we use a different custom script to label the images because the format this network uses is different. It requires labeling the position, as well as the size, of the bounding box relative to the size of the image in both x and y directions. Another difference is that we can label up to 80 classes (in our case, it was 8: for each mouse body, nose, head, and tail).

### Human Pose Detection

We used OpenPose neural network architecture to detect the human poses in the videos (Cao et al., 2017). This network uses a non-parametric representation, which is referred to as Part Affinity Fields (PAFs), to learn to associate body parts with individuals in the image. This network model is implemented in C++ and Caffe. We then use a 10 × 7 checkerboard with 115 × 115 mm square sizes to calibrate the cameras. The camera calibration and 3D pose calculations were all done in MATLAB (Mathworks, Natick, MA).

### Training the Neural Networks

We trained the networks, assessed their performance and used them for new image analysis on a computer with a TITAN X Pascal and Quadro P6000 graphics processor units (NVIDIA). The operating system was Ubuntu 16.0 with LinuxMint 18. CUDA 8.0, CUDNN 5.0 and Python 2.7 were used. On this computer, with one GPU in use, training the first network architecture (for food pellet reaching task) takes ~8 h for 600,000 iterations. Processing new images on the trained network takes ~ 50 ms per image again with one GPU. Similarly, training YOLO v3 takes ~12–14 h for 180,000 iterations, and new images are processed on the trained network at 30 frames per second with one GPU. Processing new images on OpenPose occurs at 3–4 Hz with two GPUs.

### Code to Obtain Kinematic Data

Both algorithms in mice and humans generates the positions in “.json” files. We process these files in custom written code in MATLAB (Mathworks, Natick, MA) to obtain each joint's position from each camera view. We then combine two camera views to obtain the 3D positions. We used 4 × 6 checkerboard with 4.5 × 4.5 mm square size for mouse paw videos camera calibration. After obtaining the 3D position of joints or paws, we calculate several parameters such as the velocities, trajectories, shoulder and elbow angles as well as the supination angles all with custom written MATLAB codes. All of our code is open-source and available on our GitHub page at www.github.com/aarac/DeepBehavior.

## Results

### 3D Marker-Less Paw Detection During Skilled Reaching Task

**Figure 1:**
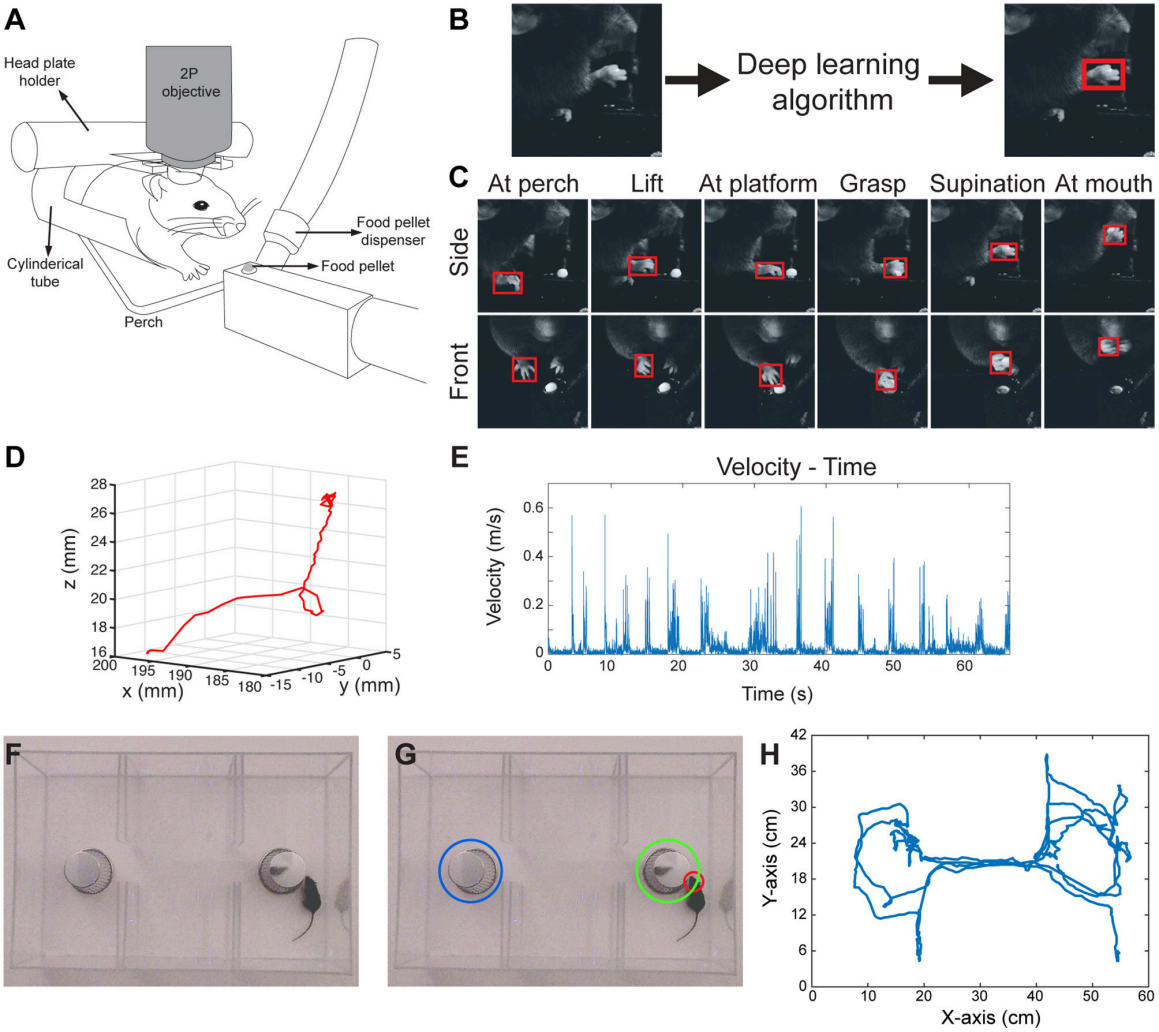
3D paw kinematics and three-chamber social behavior analysis in mice. **(A)** Schematic of skilled food pellet reaching task in head-fixed mice. This setup allows simultaneous two-photon (2P) calcium imaging or electrophysiological recordings. **(B)** An example of the data processing with feeding of raw video frames to the deep learning algorithm to obtain the coordinates of a bounding box around the paw. **(C)** Representative images showing detected paws from two camera views when the paw is in different positions. **(D)** 3D trajectory of a single reaching attempt obtained from 2D coordinates of paw positions in two camera views. **(E)** Kinematic parameters such as velocity-time graphs can be obtained from the 3D trajectories. **(F)** Raw video-frame of three-chamber test. **(G)** Representative analysis showing detection of the head of the mouse (red circle), and the cup with a stranger mouse in (green circle), and without a mouse (blue circle). **(H)** Trajectory of the mouse seen in Video-3.

Food pellet reaching in rodents is a commonly used motor behavior task to study motor learning and motor recovery (Farr and Whishaw, 2002; Guo et al., 2015). However, even simple motor behaviors such as reaching, when examined in detail, can be very difficult to define and quantify. Traditionally, performance in this task has been measured either by the percentage of the attempts in which the mouse is able to grab and eat the pellet (success rate), or with subjective scoring of each step of movement by a blinded observer (Farr and Whishaw, 2002). We have modified this task to include an apparatus for head-fixed mice to allow for future simultaneous imaging and electrophysiological recordings of the brain (Figure 1A). In this setup, the mouse is head-fixed and performs a reaching task for a food pellet. During this time, the food pellet is delivered by an automated food pellet delivery system after detection of the pellet removal (Supplementary Figure 1). During performance of the task, the animal's paw movements are video-recorded with high-speed cameras from two angles (Supplementary Figure 1). The cameras are triggered by a function generator to enable inter-camera synchronization. In order to detect the paw position in these video frames, we used a CNN model with an architecture of GoogLeNet (Szegedy et al., 2015, 2016) followed by an LSTM (long short-term memory) layer in TensorFlow (Stewart et al., 2016). This network model detects several outputs based on a set threshold value for LSTM (Supplementary Figures 2A–C). We trained this network with manually labeled images as described below. We obtained the initial weights of the GoogLeNet after training it first with ImageNet dataset. In order to manually label the images, we used a custom Python script which enabled placement of a bounding box around the paw and registered the coordinates of that bounding box in a separate text document. The input for this algorithm is the raw video frames, and the output is the coordinates of a bounding box around the right paw (Figures 1B,C; Video 1). The algorithm also provides a confidence score for each detection that can be useful for post-processing. Of note, we trained only one network with images from both front view and side view cameras. This network can detect the right paw position in both types of images.

In order to obtain 3D positions of the paw movements, we first calibrated the cameras with 24 checkerboard images (Supplementary Figures 3A,B), using a camera calibration toolbox in MATLAB (Bouguet, 2015). This toolbox creates a 3D cartesian coordinate system (Supplementary Figure 3C), which then provides the 3D position of a point when 2D positions of that point is given from the two camera views. By using this, we combined the 2D positions of paws detected by the neural network, and obtained the 3D trajectories of paw movements (Figure 1D; Video 2). After obtaining 3D coordinates, the kinematic data such as the distance traveled, time spent during the movement, maximum and average velocities can be calculated from these data (Figure 1E).

### Social Behavior Analysis in the Three-Chamber Social Interaction Test

Similar to the paw detection method, we show that the same network can be used to analyze the three-chamber social interaction test. In this test, there are three chambers that the mouse can freely explore. In one chamber there is an empty wired cup, in another chamber a wired cup with a stranger mouse inside, and the third chamber is empty (Figure 1F). The mouse can freely move and explore all three chambers. The traditional analysis measures the times spent exploring/interacting with the wired cups, as the normal mice spend more time with the cup that has the stranger mouse. In order to perform this type of analysis automatically, we detect and track the head of the mouse throughout its exploration of the chambers using the same network architecture and methods as described above. We also detect the position of the chambers and when the head of the mouse is close enough to the chambers, we count it as interaction (Figure 1G; Video 3). With this type of analysis, we can measure the interaction times with either cup automatically. Moreover, the analysis also provides the position of the animal at any given time (Figure 1H). This allows calculation of whether the mouse is moving from one chamber to another, the precise timing of interactions, interaction counts, and the mouse's velocity as it explores the chambers.

### Transfer Learning Results in Faster and More Reliable Training

**Figure 2:**
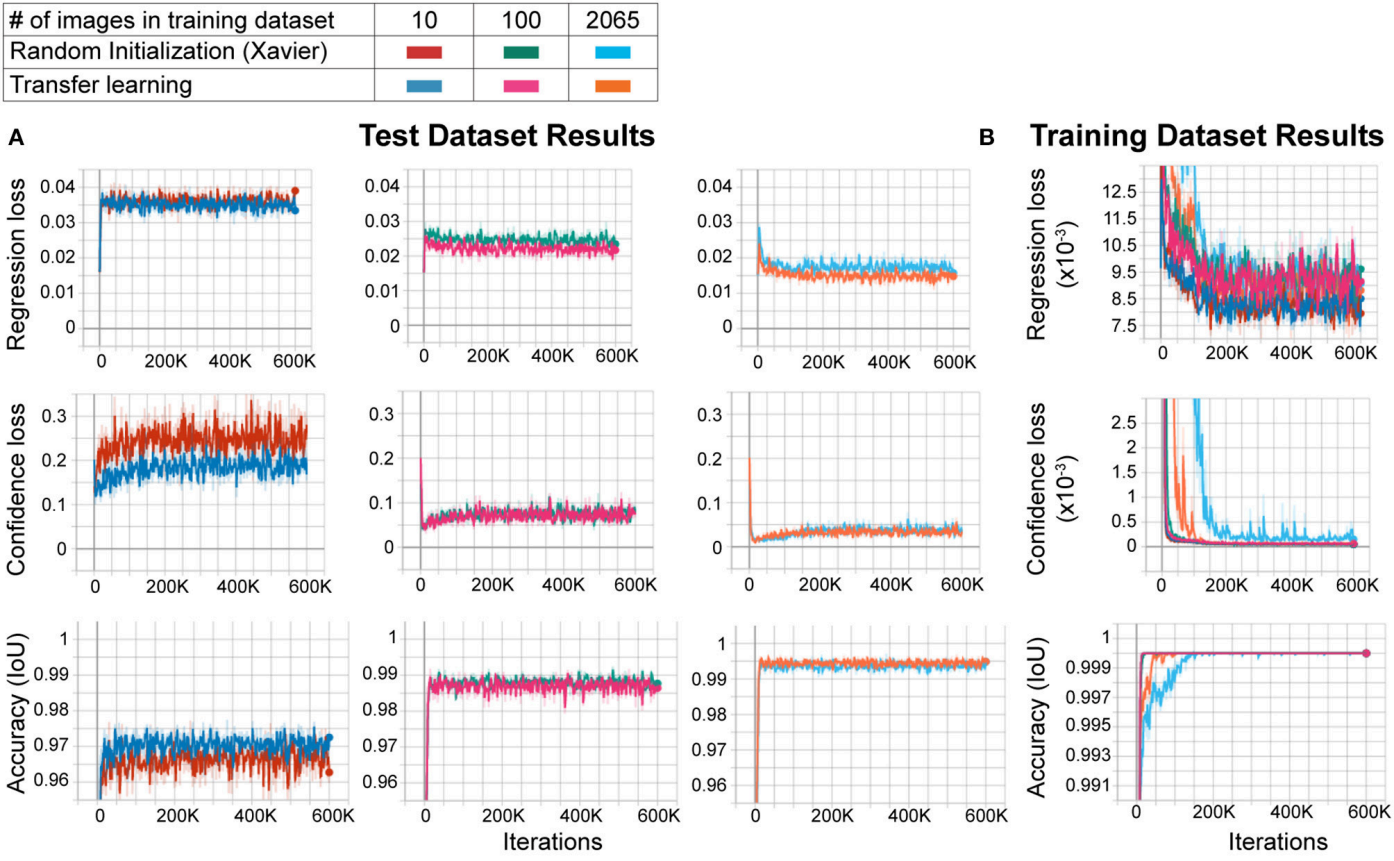
Transfer learning results in faster and more reliable training. **(A)** Training results of the test dataset. As the training dataset size increases from 10 images to 2,065 images, the regression and confidence losses decrease, and the accuracy (IoU: intersection over union) increases. For each training dataset size, transfer learning results in more accurate training compared to random initialization (low regression and confidence losses, and high accuracy). **(B)** Training results of the training dataset. Note that the transfer learning training curves in the 2,065 training dataset size group converge faster and stay stable throughout the training, meaning more reliable training.

Large datasets are required for training CNNs to obtain accurate results that generalize well. However, to create custom applications, one needs to create manually labeled training datasets from custom images. This can be challenging as labeling tens to hundreds of thousands of images manually is time-consuming and cumbersome, and defeats the purpose of creating an automated tool that should be easily modifiable. In order to overcome this, the transfer learning approach has been proposed. In this approach, the network model is first trained with another larger dataset such as ImageNet (with 1.2 million images in one thousand classes) with random initialization of the weights, followed by re-training with a smaller dataset with custom images. This method improves performance significantly (Mahajan et al., 2018). However, behavioral video recordings contain images with less variability given that they are recorded under one condition (compared to the high variability of the larger datasets such as ImageNet). Thus, the network may overfit the model when trained with random initialization. This, however, may not matter to the experimenter as it will be used to analyze only the same type of images. In fact, one (Mathis et al., 2018) of the two deep learning methods for behavior analysis in the literature uses transfer learning whereas the other (Pereira et al., 2018) does not. Thus, it is not clear whether transfer learning is really necessary to obtain good results in these types of experiments. To test whether the transfer learning approach is better with images of behavioral videos, we trained two networks with the same architecture. One of them was trained with random initialization of weights using Xavier initialization, and the other with the transfer learning approach. We used different sizes of training datasets (10, 100, and 2065 images) and the same test dataset (230 images) for each training. We found that the transfer learning approach resulted in greater accuracy (lower regression and confidence losses, and higher accuracy) with each training dataset size (Figure 2A). Moreover, as expected, increasing training dataset size improved the accuracy while decreasing confidence and regression loss (Figure 2A). As the training dataset size increased, this difference between transfer learning and random initialization decreased. However, transfer learning resulted in faster and more reliable training as evidenced by faster convergence and more stability on confidence loss and accuracy of the training dataset (Figure 2B). These results show that even with these types of behavior images with less variability, the transfer learning approach is better than training a naïve network (random initialization of weights).

**Figure 3:**
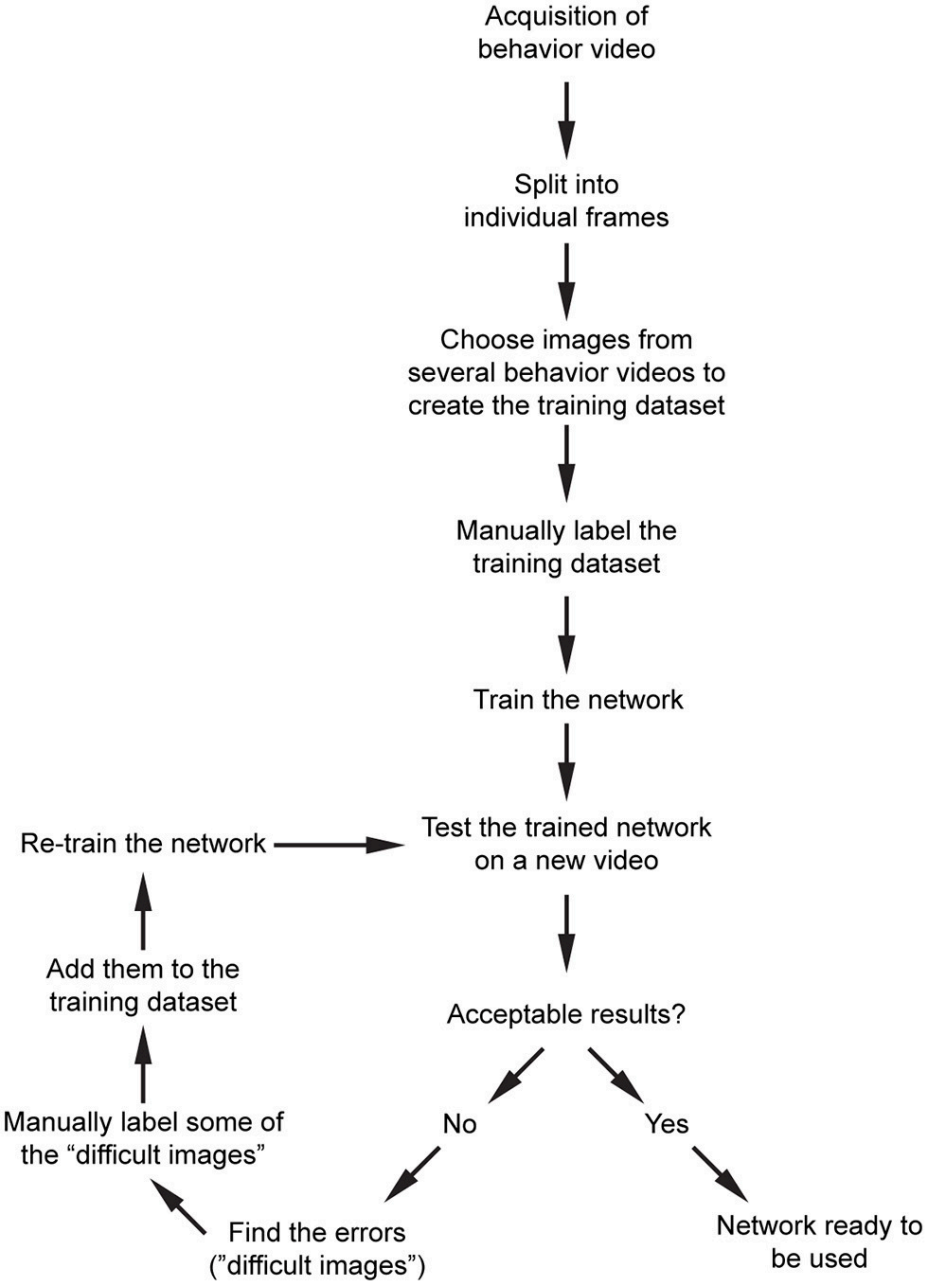
Proposed workflow for processing of the raw behavior video and training the network. After pre-processing of the videos, and initial training of the network, an iterative training algorithm allows selection of images with high variability, resulting in more generalizable training for that dataset. After the images are processed, they can be used for post-processing.

Our overall workflow is shown in Figure 3. After acquisition of behavior videos, we split them into individual frames. Next, we choose images semi-randomly based on the different positions of the mice or paws depending on the content of the videos. We then label them manually using custom scripts, and train the network that is already pre-trained with ImageNet dataset. For the above network, we recommend starting with 200–300 manually labeled images. We train the network and then test the performance on a new video. This will show what kind of errors the network makes (such as misdetection, multiple detections, etc.). We then choose some of these images where the network had a difficulty in obtaining good results, manually label them, and add them into the training dataset and retrain the network. After a few iterations, the network becomes more generalizable within that image category.

### Analysis of Social Interaction of Two Mice

**Figure 4:**
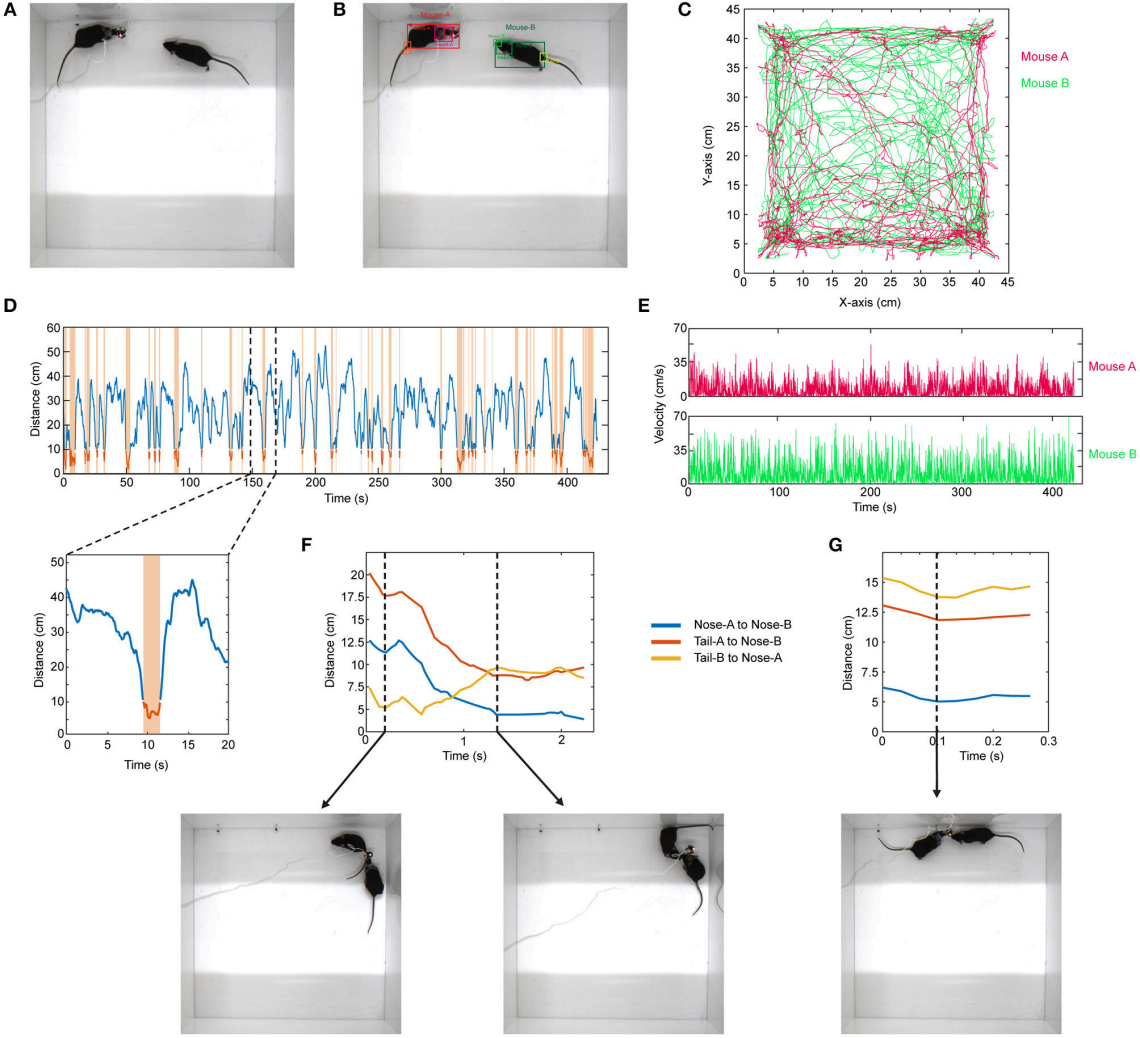
Detection of two mice separately during social interaction and post-processing of data. **(A)** Representative raw video frame when two mice are interacting in a 45 × 45 cm chamber. Of note, one of the mice has a miniscope installed on the head to do calcium imaging of neuronal activity. **(B)** Detection of two mice separately (as mouse **A** and mouse **B**) by using YOLO v3 CNN. **(C)** Trajectories of body positions of these two mice in one session of interaction (~7 min). **(D)** The distance between two mice over time during the interaction session. The time periods when two mice are critically close to each other to allow any kind of interaction are marked and highlighted by orange color. A higher magnification of one of these close contacts is shown in the lower panel. **(E)** The velocity vs. time graphs can be obtained for each mouse throughout their interaction. **(F)** A representative distance time graph over one of the close contacts. The distances are between noses, or nose and tails of two mice. In panel **(F)**, the close contact starts as mouse B sniffing mouse A's rear (shorter distance between tail-B to nose-A) but then turns into a nose-to-nose interaction. **(G)** A representative distance time graph over one of the close contacts showing a short nose-to-nose interaction between two mice.

Similar to the above example, the same approach can be expanded to the use of other network architectures. As an example, in social interaction assay, a stranger mouse is placed in a 45 × 45 cm chamber with another mouse which has a miniaturized microscope (Cai et al., 2016) (miniscope) attached to its head (Figure 4A). Their interaction is recorded from the top (bird's eye view). The interaction time between them is then recorded. This behavior assay can be powerful especially when combined with imaging of different brain regions during social behavior by using miniscopes (Cai et al., 2016). The mice can interact by sniffing nose-to-nose, nose-to-body, nose-to-tail. One difficulty in the literature has been the detection and tracking of these two mice throughout the recording. To analyze these videos, when the training dataset is created, we manually label images of the mouse without miniscope and with miniscope separately. We detect their nose, head, body, and tail (Figure 4B; Video 4). We use YOLO version-3 (Redmon and Farhadi, 2018) as the CNN architecture (Supplementary Table). This network is pre-trained with COCO dataset. After detecting the mice throughout the video, we do post-processing in MATLAB. We first separate each mouse and obtain their movement trajectories throughout the recording session (Figure 4C). We then measure the distance between their body centers, and the distance below a certain threshold is accepted as a close contact (Figure 4D). With this, we can obtain exactly when they are in close contact, the duration of contact, and their velocities throughout the recording session (Figure 4E). We then go into each “close contact” epoch and calculate the distances between each animal's nose and tail and the other animal's nose or tail. Interestingly, this gives unique interaction patterns. For example, in one close contact, mouse-A approaches mouse-B from behind (nose-to-tail interaction), but then mouse-B responds to this and turns around, and the interaction becomes nose-to-nose (Figure 4F; Video 5). In another example, the interaction is only a short nose-to-nose sniffing (Figure 4G; Video 6).

### 3D Human Pose Detection for Clinical Motor Function Assessment

**Figure 5:**
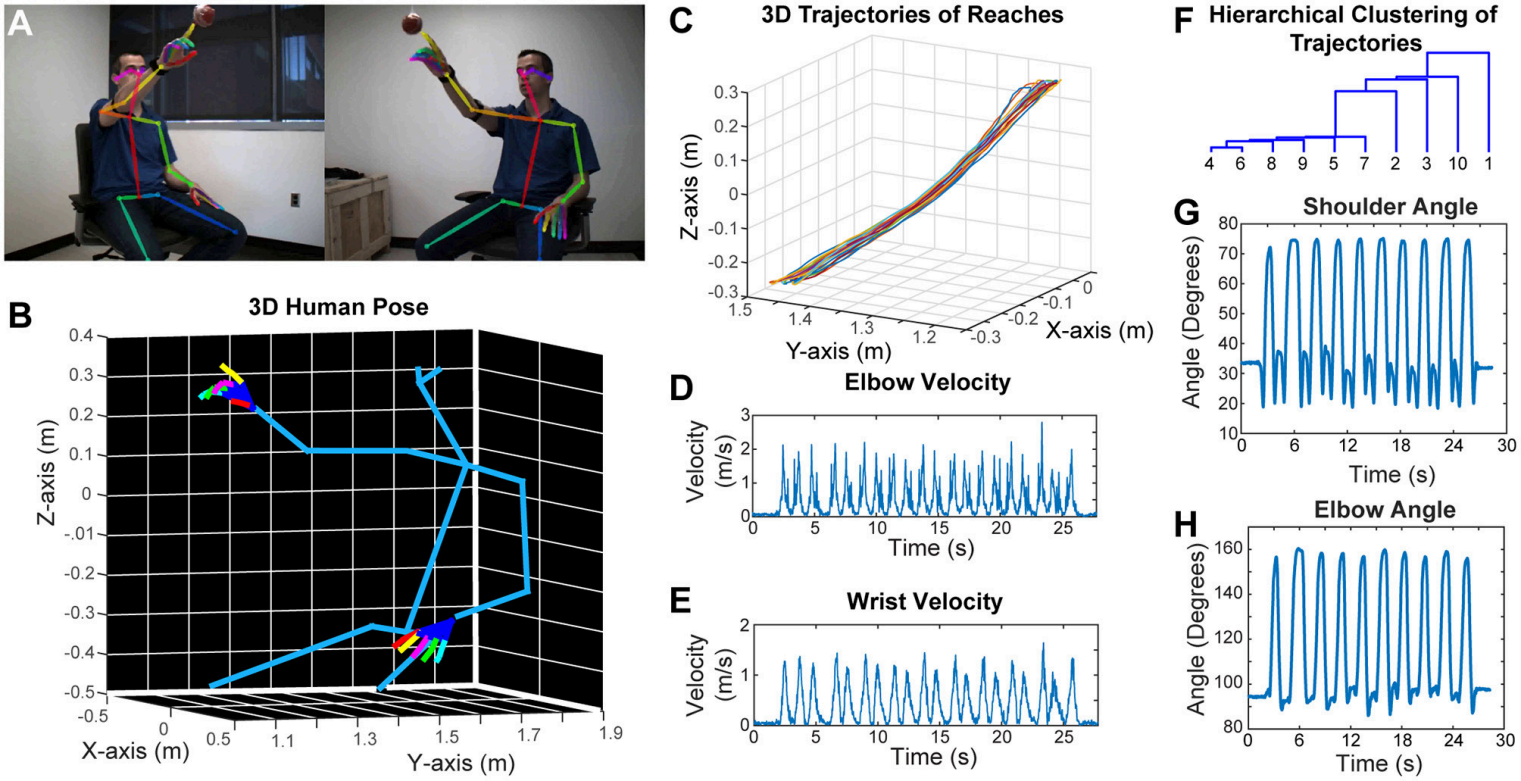
Marker-less detection of human pose with 3D kinematics. **(A)** Representative images from the stereo camera system with two cameras that have different angles, with detection of joint poses on these 2D images. **(B)** 3D model reconstructed after calibration of the two camera views in panel **(A)**, showing accurate detection of joint positions down to individual finger joints. **(C)** 3D trajectories of air reaches of the subject in panel **(A)**, there are 10 reaches with superimposed reach trajectories. Velocity vs. time graphs for right elbow **(D)** and right wrist **(E)** during these 10 reaches. **(F)** Hierarchical clustering of these 10 reaches based on the dynamic time aligned kernels of the 3D trajectories. The numbers indicate the reach number. **(G)** Shoulder vs. body angles during these 10 reaches obtained from the 3D positions. H. Elbow (arm vs. forearm) angles during the 10 reaches. All the kinematic parameters **(D,E,G,H)** were obtained from the 3D model (as seen in panel **B**).

Similar to rodent behavior analysis, the clinical motor function assessment in humans currently relies on subjective scoring of movements with ordinal scales. Performing detailed kinematic analysis in a clinical setting is challenging, and the best available techniques use robotics, exo-skeletons, sensors or externally attached markers. However, these external devices may affect the nature of the behavior. To overcome these problems, we have developed a two-camera stereo video recording system. With this system, we record the movement of the subjects at 170 frames per second, and importantly, the subjects do not need to put on any markers, or wear any sensors or special equipment. We then use a CNN (OpenPose) that was trained to detect the joint poses in humans (Wei et al., 2016; Cao et al., 2017; Simon et al., 2017) from two camera views (Figure 5A). After this, we calibrate the cameras and reconstruct the 3D models (including the individual finger joints) (Figure 5B; Video 7). As an example, we recorded a subject performing reaching movement toward a hanging ball, and then plotted the wrist movement trajectories for 10 reaches (Figure 5C), and calculated several kinematic parameters such as elbow and wrist velocities (Figures 5D,E). Moreover, after using dynamic time alignment kernels (Santarcangelo and Xiao, 2015) we can calculate the Euclidean distance between these kernels and cluster them (Figure 5F). This method identifies the similar reaches based on their trajectories in an unsupervised manner. Furthermore, from the 3D positions of joints, we can calculate the shoulder vs. body and elbow angles (Figures 5G,H).

**Figure 6:**
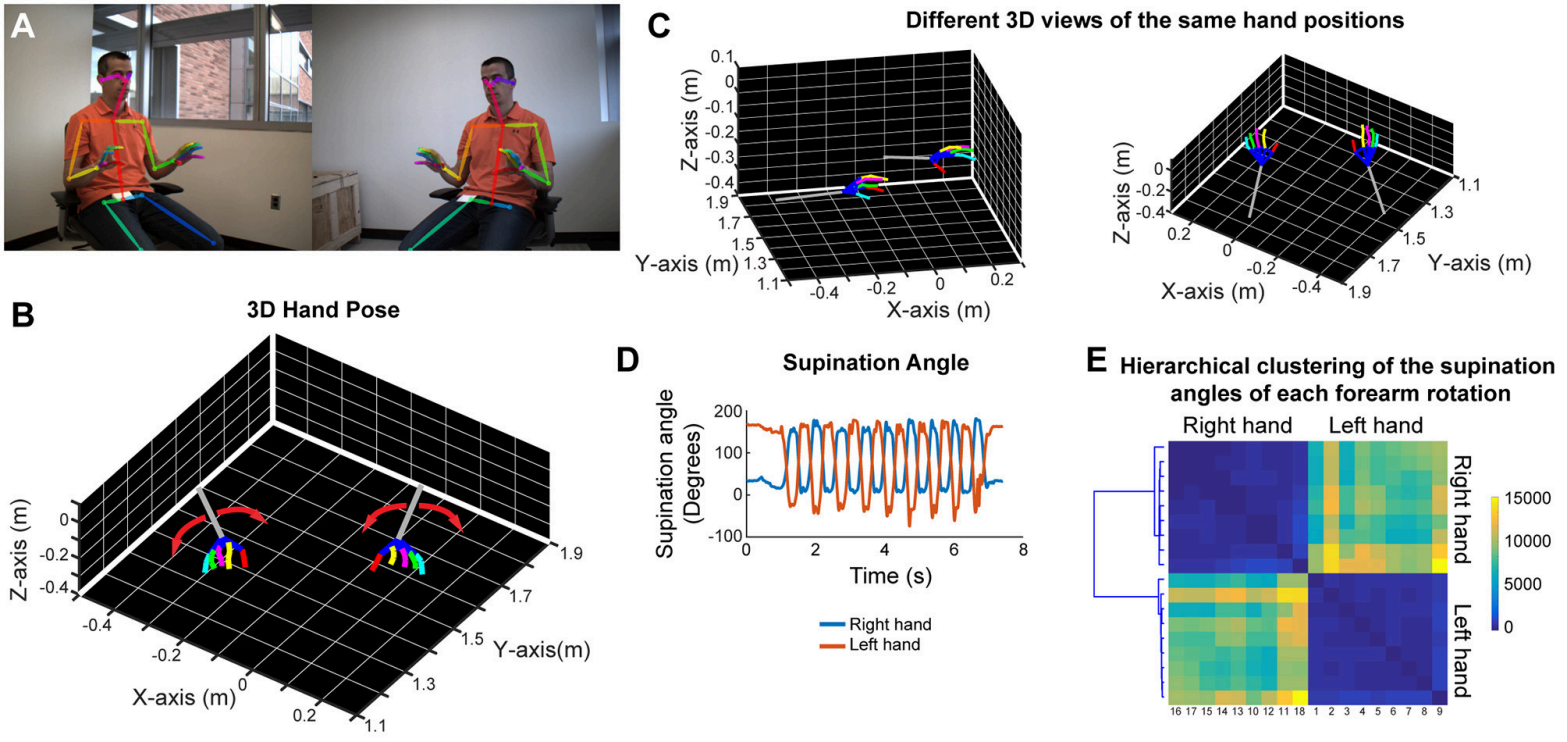
Marker-less detection of fine hand/finger movements in 3D. **(A)** Representative images from the stereo camera system, focusing more on the hand movements, with detection of the joint poses in 2D images. **(B)** Representative 3D models of both hands and forearms while the subject is doing alternating supination/pronation movements. **(C)** 3D views from different angles of the same hand positions as in panels **(A,B)**. **(D)** Supination angles (rotation angles of the forearm) of both right and left hands during nine repetitive movements. **(E)** Hierarchical clustering based on the Euclidean distance between the supination angle curves of each rotation (supination and pronation) after these curves were aligned by dynamic time warping. Please note distinct clustering of right and left hand rotations as well as the heterogeneity among rotations within each hand.

In order to analyze forearm/hand movements, we recorded the subject during an alternating supination/pronation task (Figure 6A). We can reconstruct the 3D model of the hands with individual finger joints (Figures 6B,C and Video 8). With this task, we can calculate the supination angles (rotation angle along the forearm axis) from the 3D models (Figure 6D). We then use dynamic time warping to align these supination angle curves and calculate the Euclidean distance between them. By using hierarchical clustering on these calculated Euclidean distances, we can identify similar movement patterns (Figure 6E). This analysis robustly clustered the right and left hand movements as well as the different movement patterns for each hand in a healthy subject (Figure 6E).

## Discussion

Here, we present easy-to-use methodology on how to use CNNs for behavior imaging data analysis in mice and humans. Specifically, we use three different neural network architectures and five different behavior tasks. We present methods and share tips on how to train neural networks to achieve good accuracy, and provide methods for post-processing of the data. This approach can be applied to most, if not all, of the available CNN architectures.

The transfer learning approach generally provides very good results requiring minimal number of images that need to be manually labeled for training dataset (Mahajan et al., 2018). Given the low variability of images in the videos obtained in the animal studies compared to larger datasets such as ImageNet, one argument is that overfitting may not cause significant problem given that the test images are all in the same category. However, we show that even with this type of similar image sets with low variability, the transfer learning approach makes the training faster and more reliable. Thus, the transfer learning approach should be considered for these types of analyses. The network models used in this study are chosen for their ease of use, and the same technical approach can be applied to other available network models, or any future network architecture. As the deep learning field grows and generates better and faster network architectures, those new models (or the existing ones) can be used with a similar approach.

The traditional analysis for the food pellet reaching task in rodents evaluates whether the animal can successfully grab the food pellet over a number of reach attempts (success rate). A more sophisticated method (Farr and Whishaw, 2002) breaks down this movement into different stages, and gives subjective scores based on how close they are to a predefined normal movement. However, this type of scoring system is subjective and is dependent on an evaluator watching the videos in slow motion (almost frame by frame), thus requiring significant amount of time to analyze. To overcome this, a reflective marker that is glued on the paw can be tracked (Azim et al., 2014). However, this method fails when the marker is occluded. Alternatively, traditional computer vision classifier algorithms can be used for marker-less detection of paw (Guo et al., 2015). However, these algorithms need to be trained for each video.

Deep learning applications for behavioral analysis have recently been developed (Mathis et al., 2018; Pereira et al., 2018). One (Mathis et al., 2018) of these applications uses the transfer learning approach whereas the other one does not (Pereira et al., 2018). However, these require separate training for each camera view and lack the post-processing code for kinematic analysis. While these methods are very useful, we are expanding the behavior analysis tools available for the neuroscience community.

We extend the use of same methodology into different social behavior tasks. In the three-chamber test, the traditional analysis approach has been manual measurement of interaction times with the cups (Moy et al., 2004). By using the same network architecture that we used for paw detection, we first detect the head of the mouse, and track it as the mouse explores all three chambers. This type of analysis provides more relevant information than just the interaction times, it also lowers the time spent for analysis significantly.

In the other social behavior test of two mice interacting, the traditional analysis was based on just the interaction of two mice (Kim et al., 2015). However, this analysis is very limited. Using the same transfer learning approach, but this time a different network architecture, we can automatically track two mice, one of them wearing a miniaturized microscope. Because the algorithm recognizes these mice separately (one with the miniaturized microscope, the other without), we can distinguish them even after a very close contact. The analysis also provides whether the mice are moving, and if so, their velocities, the interaction type (nose-to-nose vs. nose-to-tail sniffing), the time that they start approaching to each other, etc. This type of detailed analysis is important in identifying the details of the social interaction.

Clinical motor impairment scores (i.e., Fugl-Meyer, Action Research Arm Test) are insensitive to detect the meaningful changes in the motor function. Moreover, they may not even reflect accurate motor behavior. When tested after constraint-induced movement therapy for stroke victims, although these measurement scales showed benefit, the kinematics of how patients performed these movements didn't change, suggesting compensatory mechanisms rather than true recovery (Kitago et al., 2013). Kinematic analysis has the potential to provide information on multi-joint coordination and motor control mechanisms (Alt Murphy and Häger, 2015). Here, we demonstrate that by using video recording in a standardized way, more meaningful data with 3D kinematic parameters can easily be collected in clinical settings. The setup of the hardware is also straightforward and very portable, making it feasible to obtain data at bedside. This type of kinematic analysis reduces subjectivity by capturing whole limb movements and replacing ordinal scales with continuous ones. Moreover, this setup can be expanded in simple but meaningful ways, such as adding simultaneous electromyographic recordings in a few key muscles of interest. However, before its clinical use, one needs to perform clinimetric studies such as reliability, validity, measurement error, responsiveness to abnormal motor function, etc., but these are beyond the focus of the current study. Several kinematic metrics such as task completion time, number of movement onsets, path length ratio, number of velocity peaks, joint angles and angular velocities have been proposed to provide objective evaluation of the movement quality (de los Reyes-Guzman et al., 2014). However, more longitudinal studies are required to enable a detailed understanding of recovery patterns after injury such as stroke.

Elucidating the behavior in detail is critical to understanding the brain-behavior relationship (Krakauer et al., 2017). The tools provided here have the potential to define the behavior in more detail, and when combined with other tools to study the brain, will likely help dissect out the brain-behavior relationship. Overall, we show proof of principle of the technique using several neural network architectures and different ways of analyzing several behavior tasks in mice and humans. In the future, with the advances in the deep learning field, faster and more sophisticated methods can likely be used with the same approach.

## Ethics Statement

All animal procedures were approved by the University of California, Los Angeles, Department of Laboratory Animal Medicine Institutional Animal Care and Use Committee, and were in accordance with the AAALAC and NIH guidelines. A written informed consent was obtained from both of them prior to the recording in accordance with the Declaration of Helsinki. The consent included the use of video recordings for research, education, publication and public presentation. The protocol was approved by The UCLA Institutional Review Boards in The UCLA Office of the Human Research Protection Program (OHRPP).

## Author Contributions

AA and PG conceptualized and designed the study and wrote the manuscript with input from all authors. AA performed all the coding, implementation of the software, performed the video recordings, and analyzed the data. PZ assisted with performing video recordings, and design of the study. BD and SC helped with the design and provided guidance.

### Conflict of Interest Statement

The authors declare that the research was conducted in the absence of any commercial or financial relationships that could be construed as a potential conflict of interest.
