# Supplementary material for: DeepBehavior: A Deep Learning Toolbox for Automated Analysis of Animal and Human Behavior Imaging Data
Source: Front Syst Neurosci. 2019 May 7;13:20. doi: 10.3389/fnsys.2019.00020 (PMC6513883; doi:10.3389/fnsys.2019.00020)
Supplement: Supplementary file 1 [file Table_1.docx]

**Supplementary Data**

**DeepBehavior: A deep learning toolbox for automated analysis of animal and human behavior imaging data**

Ahmet Arac^1,*^, Pingping Zhao^1^, Bruce Dobkin^1^, S. Thomas Carmichael^1^, Peyman Golshani^1,2,3^

^1^ Department of Neurology and University of California, Los Angeles, Los Angeles, CA, United States

^2^ Semel Institute for 75 Neuroscience and Human Behavior, University of California, Los Angeles, Los Angeles, CA, United States

^3^ West Los 76 Angeles Veterans Affairs Medical Center, Los Angeles, Los Angeles, CA, United States

*** Correspondence:**

Ahmet Arac

Email: [aarac@mednet.ucla.edu](mailto:aarac@mednet.ucla.edu)

**Key words:**

Behavior analysis, deep learning, motor behavior, social behavior, human kinematics

**Supplementary Figure Legends**

**Supplementary Figure-1. Schematic of the reaching task setup from top view.**

The food pellet delivery system is automated and controlled by an Arduino board. The paw movements are recorded by synchronized cameras from two angles (front and side).

**Supplementary Figure-2. Architecture of the CNN used in Figure-1.**

A. Big picture architecture of the CNN used for paw detection and three-chamber test. A GoogLeNet architecture was followed by an LSTM layer for prediction. Adapted from Stewart et al (2016 IEEE Conference on Computer Vision and Pattern Recognition (CVPR); 2016 27-30 June 2016: 2325-2333). B. Architecture of the Inception module. C. Architecture of the GoogLeNet. B and C were adapted from Szegedy et al (2016 IEEE Conference on Computer Vision and Pattern Recognition (CVPR); 2016 27-30 June 2016: 2818-2826)

**Supplementary Figure-3. Calibration of cameras to obtain 3D coordinates.**

Checkerboard images used for calibration from side (A) and front (B) view cameras. C. 3D cartesian system created after calibration based on checkerboard images.

**Supplementary Video Legends:**

Video-1. Detection of right paw with CNN in both side and front view videos. 6x slowed.

Video-2. Reconstruction of 3D trajectory of the paw movement. 12x slowed.

Video-3. Detection of the head in three-chamber test. 2x faster.

Video-4. Detection and tracking of two mice separately during social interaction test. Normal speed.

Video-5. An example of close contact where the interaction of two mice starts with nose-to-tail, and then converts to nose-to-nose. Slowed down 2/3 of normal speed.

Video-6. An example of close contact with a brief nose-to-nose interaction. Slowed down 2/3 of normal speed.

Video-7. Detection of joint positions in a human subject performing 10 air reaches. Slowed down 5.6 times the normal speed.

Video-8. Detection of joint positions in a human subject performing 9 forearm rotations (alternating supination/pronation). Slowed down 5.6 times the normal speed.

|  | Type | Filters | Size | Output |
| --- | --- | --- | --- | --- |
|  | Convolutional | 32 | 3 x 3 | 256 x 256 |
|  | Convolutional | 64 | 3 x 3 / 2 | 128 x 128 |
|  | Convolutional | 32 | 1 x 1 |  |
| 1x | Convolutional | 64 | 3 x 3 |  |
|  | Residual |  |  | 128 x 128 |
|  | Convolutional | 128 | 3 x 3 /2 | 64 x 64 |
|  | Convolutional | 64 | 1 x 1 |  |
| 2x | Convolutional | 128 | 3 x 3 |  |
|  | Residual |  |  | 64 x 64 |
|  | Convolutional | 256 | 3 x 3 /2 | 32 x 32 |
|  | Convolutional | 128 | 1 x 1 |  |
| 8x | Convolutional | 256 | 3 x 3 |  |
|  | Residual |  |  | 32 x 32 |
|  | Convolutional | 512 | 3 x 3 /2 | 16 x 16 |
|  | Convolutional | 256 | 1 x 1 |  |
| 8x | Convolutional | 512 | 3 x 3 |  |
|  | Residual |  |  | 16 x 16 |
|  | Convolutional | 1024 | 3 x 3 /2 | 8 x 8 |
|  | Convolutional | 512 | 1 x 1 |  |
| 4x | Convolutional | 1024 | 3 x 3 |  |
|  | Residual |  |  | 8 x 8 |
|  | AvgPool |  | Global |  |
|  | Connected |  | 1000 |  |
|  | Softmax |  |  |  |

**Supplementary Table.** Architecture of YOLO version 3. Note that it has 53 convolutional layers. Adapted from arXiv:1804.02767v1 [cs.CV] 8 Apr 2018.
