# Supplementary figures and images for: DeepBehavior: A Deep Learning Toolbox for Automated Analysis of Animal and Human Behavior Imaging Data
Source: Front Syst Neurosci. 2019 May 7;13:20. doi: 10.3389/fnsys.2019.00020 (PMC6513883; doi:10.3389/fnsys.2019.00020)

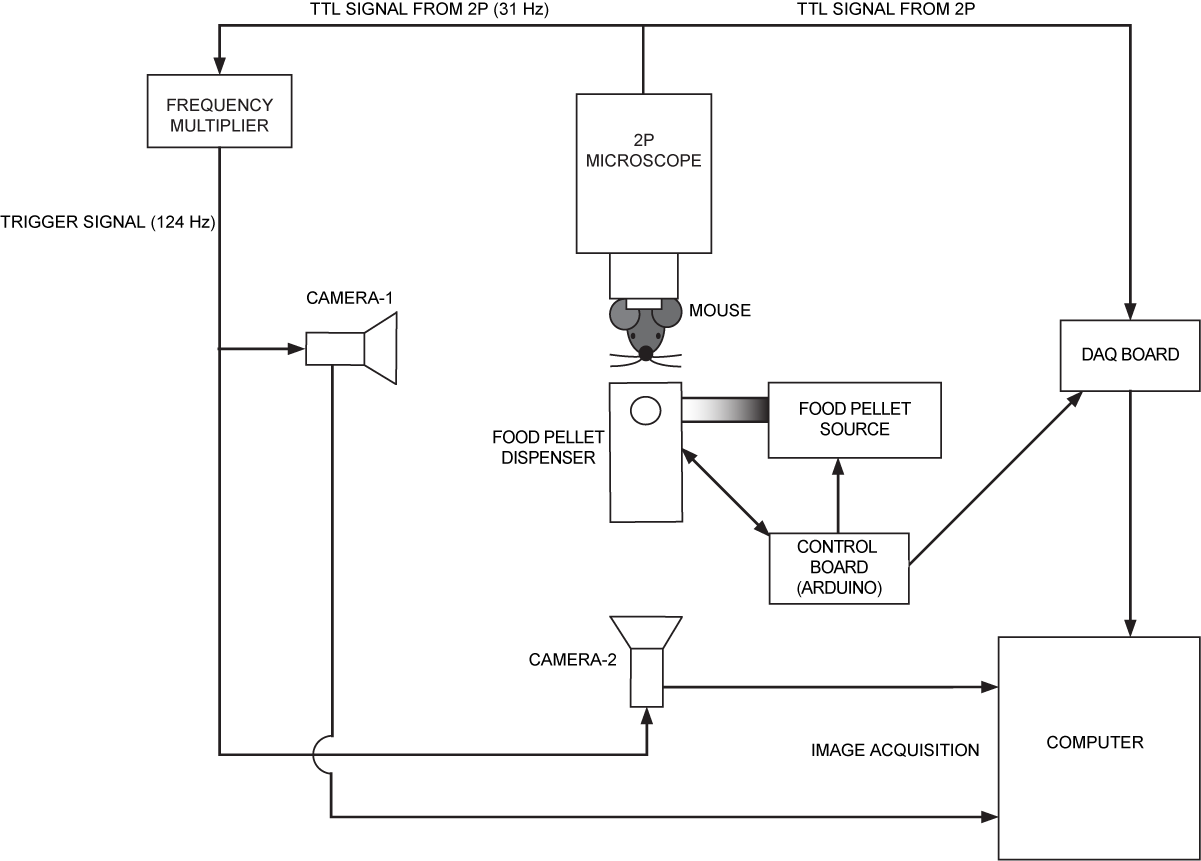

Supplement: Supplementary file 10 [file Image_1.TIF]

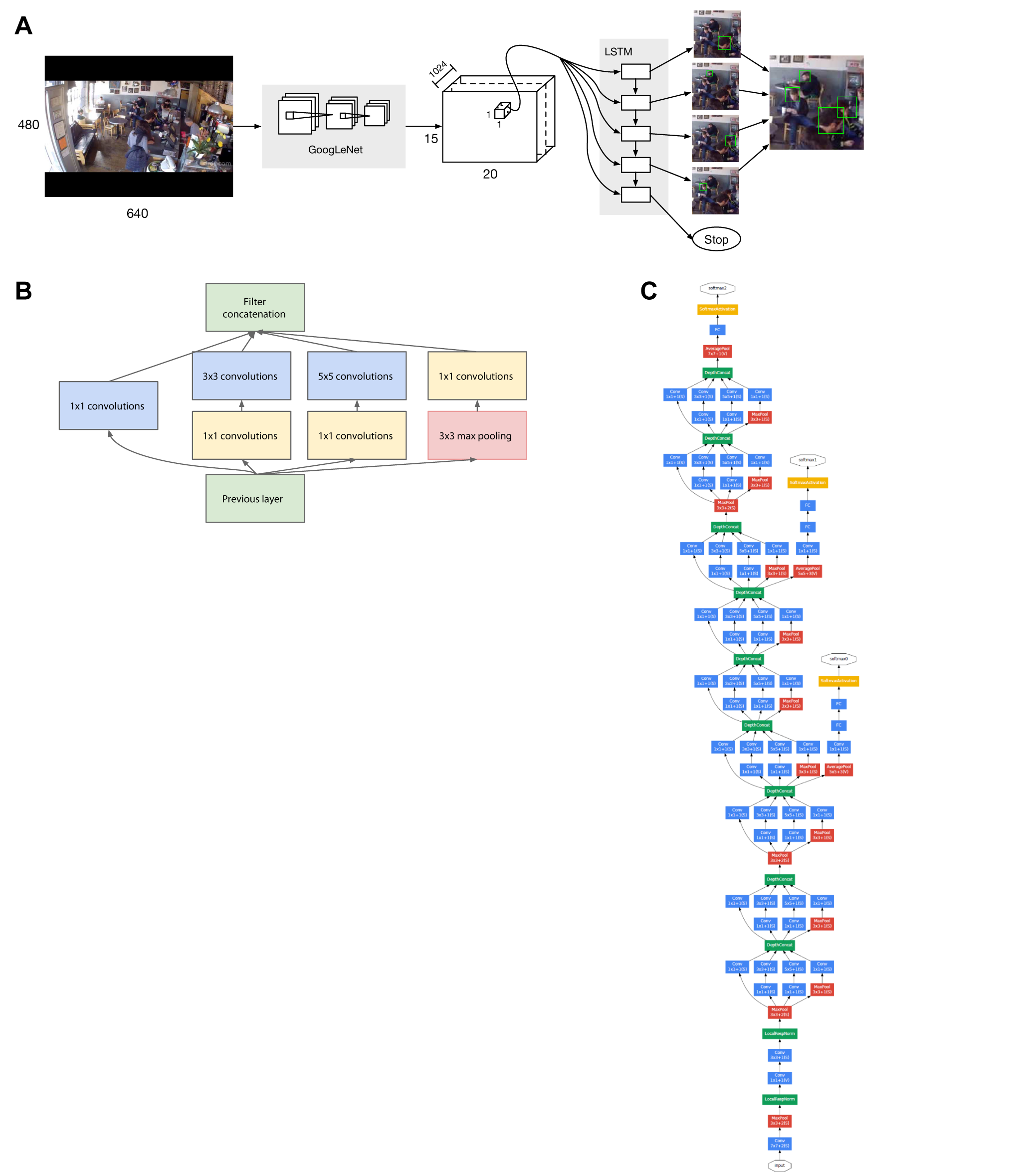

Supplement: Supplementary file 11 [file Image_2.TIF]

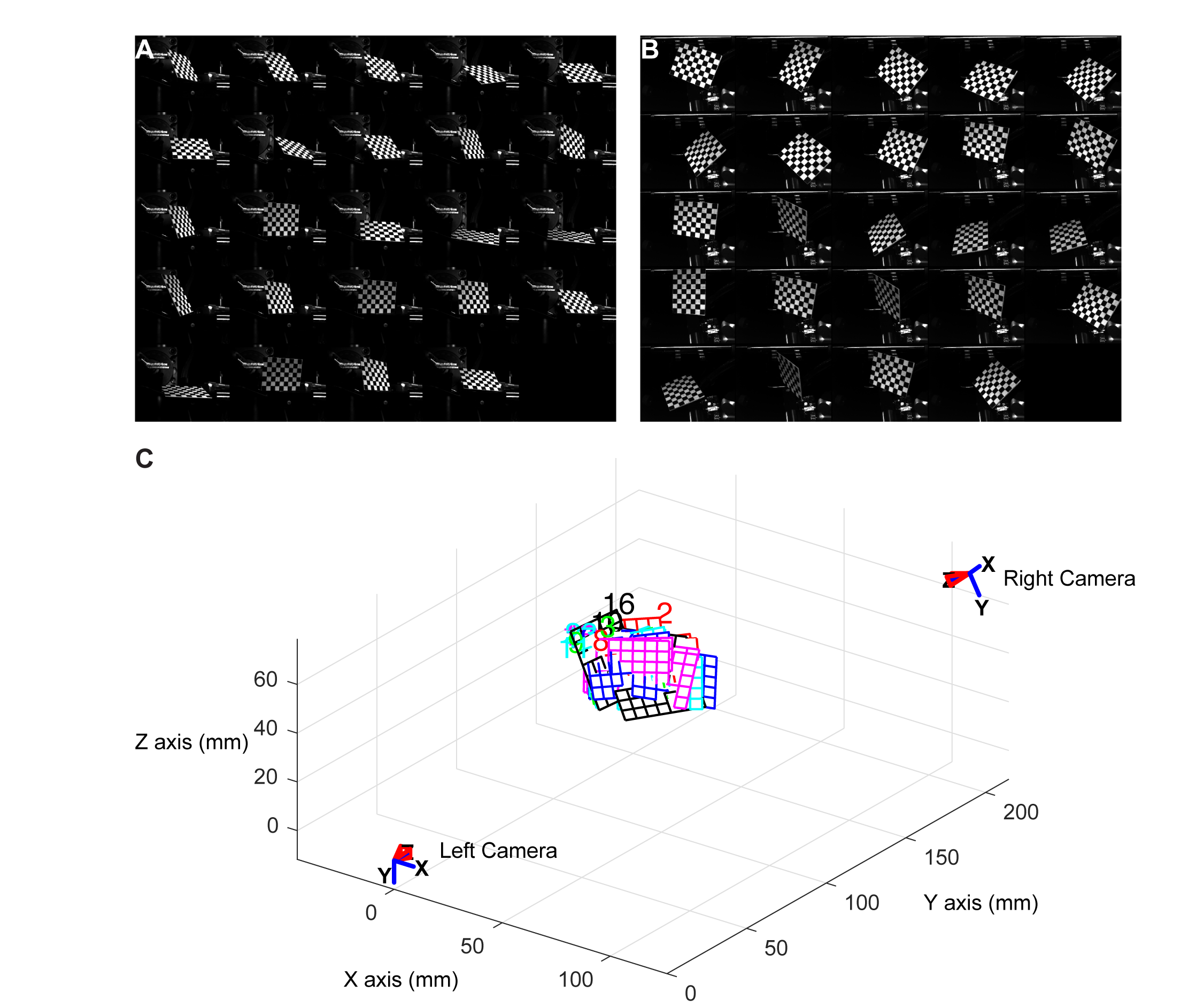

Supplement: Supplementary file 12 [file Image_3.TIF]
